# Design and development of a multichannel potentiometer for monitoring an electrode array and its application in flow analysis

**DOI:** 10.1155/S1463924602000147

**Published:** 2002

**Authors:** Jarbas J. R. Rohwedder, Celio Pasquini, Ivo M. Raimundo, Jr., M. Conceiçao, B. S. M. Montenegro, Alberto N. Araújo, Cristina M. C. M. Couto

**Affiliations:** 1 Instituto de Química UNICAMP CP 6154 Campinas CEP 13083-970 Brazil; 2 CEQUP Departamento de Química-Física Faculdade de Farmácia Porto Portugal

## Abstract

A versatile potentiometer that works with electrode arrays in flow injection and/or monosegmented flow systems is described. The potentiometer is controlled by a microcomputer that allows individual, sequential multiplexed or random accesses to eight electrodes while employing only one reference electrode. The instrument was demonstrated by monitoring an array of seven flow-through ion-selective electrodes for Ag^+^ and for three electrodes for Cl^-^, Ca^2+^ and K^+^. The figures of merit of the individual and multiplexed (summed) readings of the electrode array were compared. The absolute standard deviation of the measurements made by summing the potential of two or more electrodes was maintained constant, thus improving the precision of the measurements. This result shows that an attempt to combine the signals of the electrodes to produce a more intense signal in the Hadamard strategy is feasible and accompanied by a proportional improvement in the precision of individual measurements. The preliminary tests suggest that the system can allow for 270 determinations per hour, with a linear range from 1.0 × 10^-2^ to 1.0 × 10^-4^ mol l^-1^ for the three di¡erent analytes. Detection limits were estimated as 3.1 × 10^-5^, 3.0 × 10^-6^ and 1.0 × 10^-5^ mol l^-1^ for Cl^-^, Ca^2+^ and K^+^, respectively.


Journal of Automated Methods & Management in Chemistry
Vol. 24, No. 4 (July–August 2002) pp. 105–110

### Design and development of a multichannel potentiometer for monitoring an electrode array and its application in flow analysis

**Jarbas J. R. Rohwedder[1]*, Celio Pasquini[1], Ivo M. Raimundo Jr[1], M. Conceicão, B. S. M. Montenegro[2], Alberto N. Araújo[2] and Cristina M. C. M. Couto[2]**

[1]*Instituto de Química, UNICAMP, CP 6154, CEP 13083-970, Campinas, Brazil*

[2]*CEQUP, Departamento de Química-Física, Faculdade de Farmácia, Porto, Portugal*

*A versatile potentiometer that works with electrode arrays in flow injection and/or monosegmented flow systems is described. The potentiometer is controlled by a microcomputer that allows individual, sequential multiplexed or random accesses to eight electrodes while employing only one reference electrode. The instrument was demonstrated by monitoring an array of seven flow-through ion-selective electrodes for $Ag^+$ and for three electrodes for $Cl^-$, $Ca^{2+}$ and $K^+$. The figures of merit of the individual and multiplexed (summed) readings of the electrode array were compared. The absolute standard deviation of the measurements made by summing the potential of two or more electrodes was maintained constant, thus improving the precision of the measurements. This result shows that an attempt to combine the signals of the electrodes to produce a more intense signal in the Hadamard strategy is feasible and accompanied by a proportional improvement in the precision of individual measurements. The preliminary tests suggest that the system can allow for 270 determinations per hour, with a linear range from $1.0 \times 10^{-2}$ to $1.0 \times 10^{-4}$ mol $l^{-1}$ for the three different analytes. Detection limits were estimated as $3.1 \times 10^{-5}$, $3.0 \times 10^{-6}$ and $1.0 \times 10^{-5}$ mol $l^{-1}$ for $Cl^-$, $Ca^{2+}$ and $K^+$, respectively.*

### Introduction

Potentiometric analytical methodologies have been widely employed for implementing online measurements owing to the ruggedness and selectivity of the sensors, which can be produced for many species of interest in the field of agriculture [1], environmental chemistry [2] and clinical chemistry [3].

Ion-selective electrodes can be easily constructed, showing good analytical performance for the linear response range, selectivity, precision and lifetime. This kind of electrode is easy to miniaturize and can be arranged in arrays [4–7]. The well-established flow techniques such as flow injection analysis (FIA) [8] and monosegmented flow analysis (MSFA) [9] can also be used for sample presentation and management (dilution and conditioning) in order to expand the useful concentration range and increase sample throughput.

The goals of using an electrode array are to enhance the sensitivity and selectivity of the potentiometric methodologies and/or to perform simultaneous determinations. Sensitivity can be improved by connecting the electrodes in series [10,11], which requires the use of several reference electrodes, even though a conventional monochannel potentiometer can be used. This approach is feasible only for determination of a single species, providing an increase in the slope of the analytical curve equivalent to the sum of the slopes of each electrode employed in the array. A simple alternative to double the sensitivity in flow injection potentiometry is achieved by placing two of the same ion-selective electrode in a single-line flow injection manifold where they work alternately as the reference electrode and the indicator electrode [4]. This approach provides an analytical signal similar to the profile of a second derivative of a potentiometric titration curve and the distance between the two electrodes should be optimized to obtain maximum sensitivity. The sensitivity of potentiometric measurements can also be improved by employing multichannel instruments [4,12]. The simplest version of this kind of potentiometer allows the electronic summing of the potential of a few electrodes (two or three) and employs only one reference electrode [4].

The capabilities of such potentiometers can be further expanded to allow simultaneous determinations if the electrodes of an array can be individually accessed. Furthermore, the instrument should be versatile to allow for summing up of the responses of more than one electrode and to perform any kind of readout from an individual reading to multiplexed readings, such as those necessary for the Hadamard multiplex approach [13–15]. Most commercial potentiometers are monochannel instruments and access to the individual sensors of an electrode array cannot be made easily. Although recently some instrument manufacturers have offered multichannel instruments, the associated software is not sufficiently flexible to allow full user-tailored data acquisition [16]. Some contributions have been described in the literature for the development of multichannel potentiometers. Couto *et al.* [4] described a three-channel summing circuit for increasing the sensitivity of the potentiometric measurements, although the electrodes cannot be individually accessed. Alexander *et al.* [12] developed a multichannel circuit that can monitor up to six analytes simultaneously, but which employs a reference electrode for each indicator electrode. Forster and Diamond [6] used a four-channel potentiometer to perform non-linear calibration of ion-selective electrode arrays, where one of the electrodes should contain the ionophores of the three ISE. Although these contributions can either improve the sensitivity of the measurements [12] or perform simulta-

* To whom correspondence should be addressed.

neous determinations [6], they do not provide random access to the electrodes or multiplexing reading, which is necessary for the Hadamard multiplexed approach.

This paper describes a multichannel potentiometric instrument, which is versatile to permit data acquisition in any user-selected form. The instrument has been evaluated for its ability to sum up the potential produced by many electrodes selective to the same analyte ($Ag^+$) and to access the individual reading of an array containing three selective electrodes for $Cl^-$, $Ca^{2+}$ and $K^+$. An evaluation of the instrument was made for operation with FIA and MSFA manifolds and for individual (sequential/random) or Hadamard multiplexed measurements.

### Materials and methods

### *Reagents*

All reagents were of analytical grade and the solutions were prepared by using distilled and deionized water. The $Ag^+$ solutions in the $1.0 \times 10^{-1}$ to $1.0 \times 10^{-4}$ mol $l^{-1}$ concentration range were prepared by diluting a 1.0 mol $l^{-1}$ $AgNO_3$ stock solution. Solutions containing $K^+$, $Ca^{2+}$ and $Cl^-$ ions in the $1.0 \times 10^{-1}$ to $1.0 \times 10^{-5}$ mol $l^{-1}$ concentration range were also prepared by proper dilution of 1.0 mol $l^{-1}$ $KNO_3$, $Ca(NO_3)_2$ and NaCl stock solutions. The ionic strength of all solutions was adjusted to 1.0 mol $l^{-1}$ with a $NaNO_3$ solution.

### *Multichannel potentiometer*

Figure 1 shows the electronic layout of the multichannel potentiometer constructed. The electronic circuit of the instrument is very simple and has been conceived to operate with up to eight selective electrodes and only one reference electrode. Each indicator electrode is connected to a high-impedance FET operational amplifier (LM348) mounted in a buffer configuration. The selection of the electrode (or electrodes) that will produce the signal is made through two four-input analogue switches (ADG 201A). The control of the switches is made by a computer interface (PCL-711S, Advantech), through eight digital outputs. The signal (or the sum of the signals if more than one electrode is simultaneously connected) is sent to the inverter input of a high-impedance operational amplifier (CA3140). The reference electrode is connected to the non-inverter input of this operational amplifier. When only one electrode is connected, the circuit works as a comparator. The same circuit acts as a summing point, adding the electrochemical potential differences generated by more than one electrode when they are simultaneously selected to participate in the measurement of a single species.

### *Flow system*

Figure 2 shows a schematic diagram of the flow system employed. The system employs a four-channel peristaltic pump (Ismatec MS-Reglo) and a proportional injector

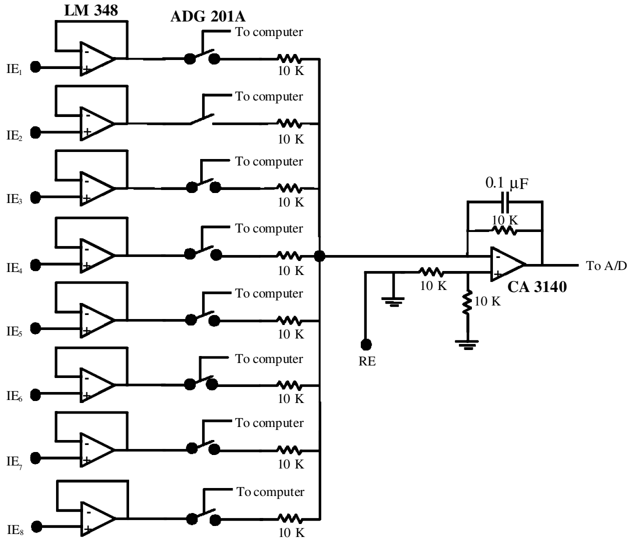

*Figure 1. Electronic circuit of the multichannel potentiometer.*

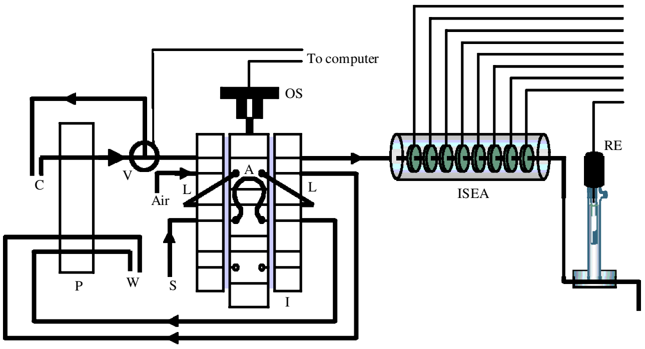

*Figure 2. Schematic diagram of the flow manifold. C, carrier fluid; P, peristaltic pump; V, three-way solenoid valve; L1/L2, air loops; A, sample loop; S, sample; OS, optical switch; ISEA, ion-selective electrode array; RE, reference electrode; W, waste.*

[17] to insert the sample into a MSFA or into a FIA (without the air loops, $L_1$ and $L_2$) manifold. An opto-switch (OS) is coupled to the sliding bar of the injector in order to flag each sample injection for the computer. The manifold includes a three-way solenoid valve (NResearch), placed before the proportional injector and used to deviate the carrier flow for stopped flow measurements. The electrode array was placed 10 cm from the injection port. The reference electrode was a standard double junction calomel (Corning — 900200), filled with a 1.0 mol $l^{-1}$ $NaNO_3$ external solution and placed after (at 1.0 cm) the electrode array. Tygon pumping tubes and polyethylene conducting tubes (0.8 mm i.d.) were employed throughout. The carrier solution was always a 1.0 mol $l^{-1}$ $NaNO_3$ solution.

### *Electrode arrays*

Two electrode arrays were employed in the present study. The first was used to investigate the performance of the potentiometer regarding its capability of adding the electrochemical potentials generated by each indicator electrode. The array contained seven silver sulphide flow-through electrodes with internal diameters of

1.0 mm [18]. These 1.0 mm-thick electrode discs were arranged in series inside acrylic rods having a 15 mm external diameter and spaced from each other by 4.0 mm. The other array was built with three different membrane-selective electrodes for $K^+$, $Ca^{2+}$ and $Cl^-$. The metal ion-selective electrodes were based on polymeric liquid membranes, constructed as described [19, 20], while the chloride-selective electrode was constituted by an $AgCl/Ag_2S$ solid membrane [18]. This array was employed to evaluate the multiplexing and sequential reading capabilities of the potentiometer.

### *Computer and software resources*

The multichannel potentiometer was controlled by an IBM-compatible 300 MHz PC-486 furnished with a PCL-711S (Advantech) parallel interface. This interface has a 12-bit analogue-to-digital converter with a conversion rate of 100 kHz. Eight digital outputs of the interface were employed for electrode selection.

Software written in Microsoft VisualBasic 5.0 was developed for electrode selection and acquisition, treatment and the storage of data.

### Results and discussion

### *Evaluation of the potentiometer with the $Ag^+$ electrode array*

The potentiometer constructed was evaluated for its capability of random access to any electrode of the array of seven silver sulphide electrodes and to verify the performance of the summing circuit. In this case, solutions containing silver ions from $1.0 \times 10^{-1}$ to $1.0 \times 10^{-5}$ mol $l^{-1}$ were continuously pumped at 4.0 ml $min^{-1}$ through the electrode array. Usually the responses of the electrodes are slightly different from each other, this behaviour being explained based on the process of manufacturing and maintenance of the solid membranes. Hence, of the seven electrodes, two presented a Nernstnian response (59.6 mV per decade of concentration), three showed a sub-Nernstnian response and two a super-Nernstnian response (table 1).

The response produced when more than one electrode was connected to the summing circuit showed that the individual responses are additive, independent of the number and of which electrodes were selected to comprise the final potentiometric signal. Figure 3 shows the signal as a function of the number of the electrodes that participate in the measurement; the straight line obtained presents a slope of 400.5 mV, very close to the sum (404.5 mV) of the individual slopes listed in table 1. This result also demonstrates that the electronic circuit of the multichannel potentiometer does not distort the signal when the electrical potentials of the indicator electrodes are summed, in accordance to the results obtained by Couto *et al.* [4] for summing two electrodes. In addition, the linearity of the response as a function of the concentration of $Ag^+$ ions is maintained from $10^{-4}$ up to $10^{-1}$ mol $l^{-1}$, with a regression coefficient of 0.9996.

*Table 1. Results obtained in a continuous flow system for the calibration of the silver-selective electrodes in the concentration range from $1.0 \times 10^{-5}$ to $1.0 \times 10^{-1}$ mol $l^{-1}$.*

| Electrode | Slope (mV/pAg) | Linear coefficient (mV) | Correlation |
|---|---|---|---|
| 1 | 53.0 | 425 | 0.9998 |
| 2 | 54.0 | 437 | 0.9991 |
| 3 | 61.0 | 459 | 0.9999 |
| 4 | 58.5 | 448 | 0.9999 |
| 5 | 48.0 | 409 | 0.9981 |
| 6 | 65.5 | 460 | 0.9951 |
| 7 | 64.5 | 449 | 0.9962 |

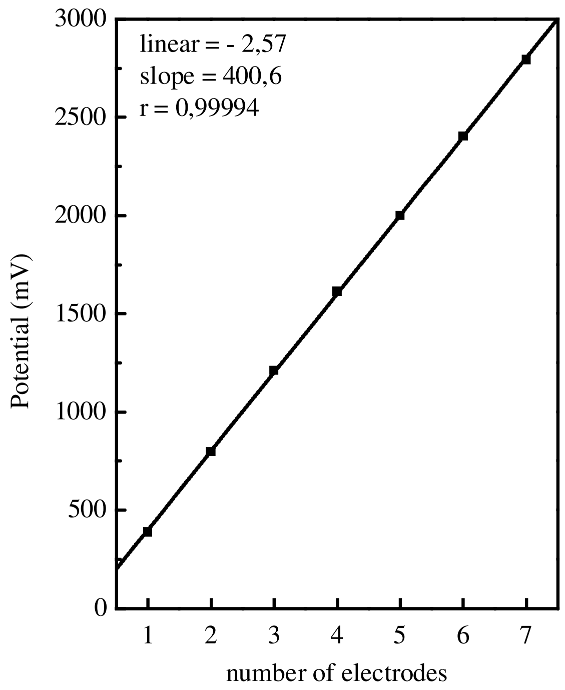

*Figure 3. Potential observed as a function of the number of silver electrodes used in the measurement for a $1.0 \times 10^{-1}$ mol $l^{-1}$ $Ag^+$ solution.*

### *Evaluation of the potentiometer with the $K^+$, $Ca^{2+}$ and $Cl^-$ electrode array*

This array was evaluated for use with FIA and MSFA systems. In this case, the sampling rate of the signals produced by the different indicator electrodes must be high enough to allow the sequential monitoring of the sensors. The sampling rate was determined by the time necessary for the sequential switch to move from one to another electrode and by the time necessary to read the potentiometric signal. The analogue switch, the interface board and computer control software employed allowed a minimum switching time of 100 μs. It means that about 10 000 measurements of any individual electrode can be performed per second. For an array of eight electrodes, data can be acquired for each electrode at 1250 $s^{-1}$. Note that the sampling rate provided by the circuitry and computer is fast enough when compared with the flow parameters and electrode responses, which, therefore, are the limiting factors of sample throughput.

*Table 2. Results obtained in the FIA and MSFA systems for the calibration of $K^+$, $Ca^{2+}$ and $Cl^-$ electrodes in the concentration range from $1.0 \times 10^{-5}$ to $1.0 \times 10^{-1}$ mol $L^{-1}$.*

| | FIA | | | MSFA | | |
|---|---|---|---|---|---|---|
| | $K^+$ | $Ca^{2+}$ | $Cl^-$ | $K^+$ | $Ca^{2+}$ | $Cl^-$ |
| Slope (mV/pIon) | 51 | 24 | −54 | 52 | 23 | −52 |
| Linear coefficient (mV) | −125 | 21 | −100 | −128 | 18 | −96 |
| LOD (mol $l^{-1}$) | $1.0 \times 10^{-5}$ | $3.0 \times 10^{-6}$ | $3.1 \times 10^{-5}$ | | | |
| Correlation | 0.9994 | 0.997 | 0.998 | 0.9996 | 0.993 | 0.999 |

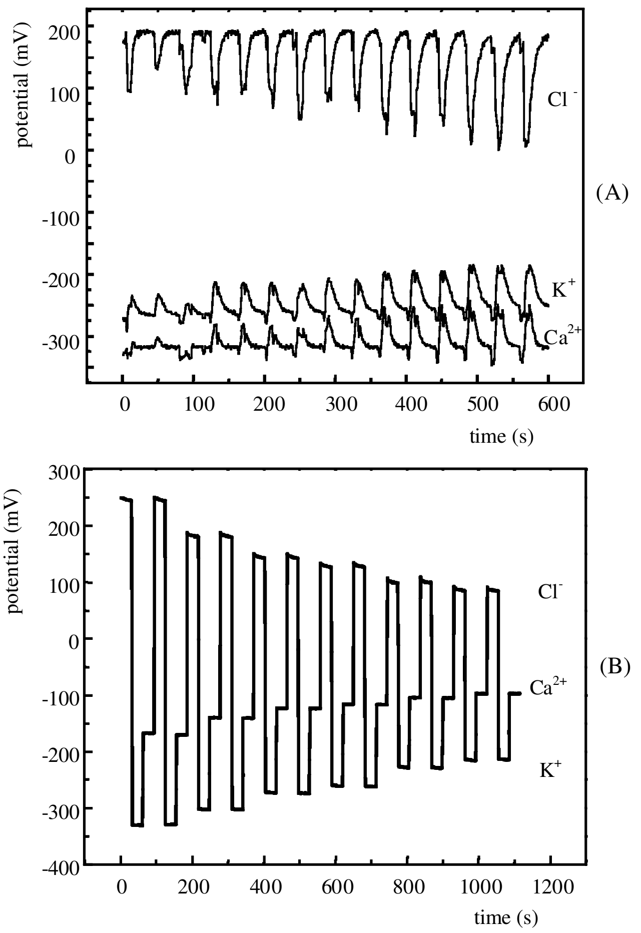

*Figure 4. Profile of the signal obtained for $Cl^-$, $Ca^{2+}$ and $K^+$ electrodes in (A) FIA system and (B) MSFA system.*

It has been demonstrated that the washing time for the flow-through electrodes is decreased by employing higher carrier flow rates in the system, as the surface of the electrode is cleaned mainly due to diffusion effects, as a consequence of the laminar flow usually found in these systems [4]. Therefore, values around 8.0 ml $min^{-1}$ have been employed for both the FIA and the MSFA approaches. The multichannel potentiometer can deal with such a high flow rates without losing information coming from the electrodes of the array. In fact, both the FIA and the MSFA signals can be fully recovered at the sampling rate achieved by the instrument for all three indicator electrodes, as can be noted in figure 4.

The signals shown in figure 4 were obtained by the injection of 500 μl reference solutions containing the three ions. Typical signals, obtained by sequential reading of the electrodes, showed no significant difference from those obtained by reading only one electrode each time for both the FIA and the MSFA systems. In the MSFA system, the air bubbles restrict the sample dispersion and the measurements resemble those of a conventional batch procedure, showing a staircase profile. The sequential measurements of the electrodes started when the sample monosegment reaches the reference electrode (detected by a regular potential reading for the last electrode in the array), ensuring that the array and reference electrode are in electrical contact through the monosegment solution. Table 2 shows the results obtained for $K^+$, $Ca^{2+}$ and $Cl^-$ ions by employing the FIA and the MSFA systems.

The MSFA approach presented no significant advantage over the FIA system in the present case as the sensitivity (and even the washing time) was not improved. However, MSFA can present some advantage for sensors with lower response times as the flow can be easily stopped (by switching valve V in figure 2) when the sample monosegment is inside the system, and a long enough waiting time can be used to attain a suitable response. The MSFA approach can also facilitate standard addition procedures commonly employed to overcome matrix effects in potentiometry, as the same sample aliquot can be employed in this process [21].

The sample throughput for both flow systems was about 90 $h^{-1}$. As three sensors were monitored after one sample introduction, the number of determinations were 270 $h^{-1}$. A system with an array of eight electrodes for eight different analytes will perform 720 determinations per hour, without any signal distortion, ensured by the higher sampling rate of the multichannel potentiometer.

### *Preliminary evaluation of the potentiometer for Hadamard multiplexed reading*

The Hadamard approach for data acquisition requires the electrode readings to be combined in a pre-established way guided by a Simplex matrix [22–24]. The individual signals can be recovered after a mathematical transformation (Hadamard Transform or demultiplexation). The signals thus obtained present a gain in the signal-to-noise ratio known as multiplex gain, which is equal to the square root of two to a power determined by the number of electrodes minus one, divided by two. However, this gain is attained only if the noise remains constant and is limited by the sensor that produces the multiplexed signal [22]. Recently, it has been demonstrated that voltammetric signals obtained with the Hadamard approach from an array of ultra-microelectrodes show a gain in the signal-to-noise ratio within the expected value predicted by the multiplex theory. The multiplex strategy has not been evaluated for the potentiometric electrochemical measurements made with arrays of electrodes.

A preliminary evaluation of the potentiometer was carried out, considering its further application to perform Hadamard multiplexed reading of the electrodes in the array. The potentiometer constructed allows connecting the electrodes to produce the multiplexed reading of a Hadamard matrix of order $2^n - 1$ (here $n = 2$) [22]. The lines of the Hadamard multiplex matrix would be (1 1 0), (1 0 1) and (0 1 1). The digit (1) means that the electrode participates (is connected) in the total multiplexed signal measured, while (0) means that the electrode is not connected. Then, the three multiplexed signals obtained under computer control would be submitted to a Hadamard transform mathematical operation (demultiplex) [22], regenerating the individual responses for each sensor. Note that when using the Hadamard approach, three multiplexed reading measurements are made and each electrode is read twice. It has been demonstrated that this approach results in a better limit of detection for spectrophotometric [25] and voltammetric [14] measurements. In addition, the multiplexed reading presents the Felgett gain, which can predicts that $(N - 1)$ measurements of the same sensor are performed in the same time interval of a single measurement of a sequential reading of an array of $N$ sensors. This gain results in a better precision of the response produced for each sensor, considering the same interval of time for measurements.

To mimic the Hadamard multiplexed measurements, three chloride electrodes were evaluated regarding the limit of detection and precision. Table 3 lists the results obtained with a single electrode and those obtained by summing the potential of these three electrodes. The limit of detection, determined according to the IUPAC recommendations for selective electrodes [26], does not show any improvement when the responses of the electrodes are summed up by the instrument. This behaviour was expected as the detection limits in potentiometric sensors are defined by the chemical equilibrium associated to the species that participate in the membrane composition and with the response mechanism, instead of instrumental (electronic) parameters. However, the precision of the measurements for concentrations above the detection limit is improved due the increase in the potentiometric signal when the response of more than one electrode are added. As the potential value has increased due to the sum, the relative standard deviation has been improved. Considering the behaviour of the signal precision as a function of the number of electrodes employed in a measurement, it is possible to predict the use of the multichannel potentiometer in more complex measurement protocols, as in the Hadamard multiplexed approach. This type of measurement would, at least, ensure an increase in the precision of individual readings

*Table 3. Limits of detection for $Cl^-$ electrodes, considering individual measurements and the sum of their responses.*

| Electrode | Blank signal (mV) | sd (mV) | LOD (mol $L^{-1}$) |
|---|---|---|---|
| 1 | 211 | 2 | $3.1 \times 10^{-5}$ |
| 2 | 206 | 3 | $2.3 \times 10^{-5}$ |
| 3 | 210 | 2 | $2.4 \times 10^{-5}$ |
| 1 + 2 + 3 | 496 | 2 | $1.7 \times 10^{-5}$ |

of each electrode, without increasing the measurement time, as previously observed for voltammetric measurements [14].

### Conclusions

The multichannel potentiometer proposed here is a versatile, low-cost instrument that can improve potentiometric measurements. It allows one to access arrays of electrodes at a rate high enough for the most common flow analysis techniques such as FIA and MSFA. The number of determinations increases with the number of ion-selective electrodes in the array times the sampling frequency allowed by the flow system, reducing, as a consequence, sample consumption. In the present work, a sampling frequency of $270\,h^{-1}$ was achieved, as three selective electrodes were used in the array and 90 samples were processed per hour.

Besides these advantages, the instrument permits the grouping of two or more selective electrodes for the same analyte, improving the signal magnitude for that species, as well as Hadamard multiplexed data acquisition. The adoption of this last approach would result in an additional gain in the precision of the potentiometric measurements.

### Acknowledgements

Authors are grateful to ICCTI (Portugal) and CAPES (Brazil) for financial support and to Professor C. H. Collins for the English revision of the manuscript.

### References

1. Silva, F. V., Nogueira, A. R. A., Souza, G. B. and Cruz, G. M., *Analytical Science*, **16** (2000), 361.
2. Martinez-Barrachina, S., Alonso, J., Matia, L., Prats, R. and del Valle, M., *Analytical Chemistry*, **71** (1999), 3684.
3. Georgiou, M. E., Georgiou, C. A., Koupparis, M. A., *Analytical Chemistry*, **71** (1999), 2541.
4. Couto, C. M. C. M, Lima, J. L. F. C. and Montenegro, M. C. B. S. M., *Analytical Sciences*, **13** (1997), 403.
5. Fernandes, J. C. B., Oliveira Neto, G., Rohwedder, J. J. R. and Kubota, L. T., *Journal of the Brazilian Chemical Society*, **11** (2000), 349.
6. Forster, R. J. and Diamond, D., *Analytical Chemistry*, **64** (1992), 1721.
7. Dimitrakopoulos, L. T. and Dimitrakopoulos, T., *Electroanalysis*, **13** (2001), 161.
8. Ruzicka, J and Hansen, E. H., *Analytica Chimica Acta*, **78** (1975), 145.

9. Pasquini, C. and Oliveira, W. A., *Analytical Chemistry*, **57** (1985), 2575.
10. Borzitsky, J. A., Dvinin, A., Petrukhin, O. M. and Urusov, Y. I., *Analyst*, **118** (1993), 859.
11. Suzuki, K., Tohda, K. and Shiari, T., *Analytical Letters*, **20** (1987), 1773.
12. Alexander, P. W., Di Benedetto, L. T., Dimitrakopolous, T., Hibbert, D. B., Ngila, J. C., Sequeira, M. and Shields, D., *Talanta*, **43** (1996), 915.
13. Rohwedder, J. J. R. and Pasquini, C., *Analyst*, **123** (1998), 1641.
14. Rohwedder, J. J. R. and Pasquini, C., *Analyst*, **123** (1998), 1861.
15. Freire, R. S., Rohwedder, J. J. R. and Pasquini, C., *Analyst*, **124** (1999), 1657.
16. Shatkin, J. A., Brown, H. S. and Licht, S., *Analytical Chemistry*, **67** (1995), 1147.
17. Bergamin F$^o$, *H., Medeiros, J. X., Reis, B. F.* and Zagatto, E. A. G., *Analytica Chimica Acta*, **101** (1978), 9.
18. Ferreira, I. M. P. L. V. O., Lima, J. L. F. C., Rocha, L. M. S., *Fresenius Journal of Analytical Chemistry*, **347** (1993), 314.
19. Alonso-Chamarro, J., Bartrolí, J., Jun, S., Lima, J. L. F. C., Montenegro, M. C. B. S. M., *Analyst*, **118** (1993), 1527.
20. Pimenta, A. M., Araújo, A. N. and Montenegro, M. C. B. S. M., *Journal of Pharmaceutical and Biomedical Analysis* (submitted).
21. Aquino, E. V., Rohwedder, J. J. R. and Pasquini, C., *Analytica Chimica Acta*, **438** (2001), 67.
22. Busch, W. K. and Busch, M. A., in J. D. Winefordner (ed.), *Multielement Detection Systems for Spectrochemical Analysis* (New York: Wiley, 1990), 170.
23. Ibbett, R. N., Aspinall, D. and Grainger, J. F., *Applied Optics*, **7** (1968), 1089.
24. Nelson, E. D. and Freedman, M. L., *Journal of Optical Society of America*, **60** (1970), 1664.
25. Poppi, R. J., Vazquez, P. A. M. and Pasquini, C., *Applied Spectroscopy*, **46** (1992), 1822.
26. Pungor, E., *Pure and Applied Chemistry*, **64** (1992), 503.

